# The Role of Gut Microbiota Dysbiosis in Erectile Dysfunction: From Pathophysiology to Treatment Strategies

**DOI:** 10.3390/microorganisms13020250

**Published:** 2025-01-23

**Authors:** Aris Kaltsas, Ilias Giannakodimos, Eleftheria Markou, Konstantinos Adamos, Marios Stavropoulos, Zisis Kratiras, Athanasios Zachariou, Fotios Dimitriadis, Nikolaos Sofikitis, Michael Chrisofos

**Affiliations:** 1 Third Department of Urology, Attikon University Hospital, School of Medicine, National and Kapodistrian University of Athens, 12462 Athens, Greece; ares-kaltsas@hotmail.com (A.K.); iliasgiannakodimos@gmail.com (I.G.); constantinos.adamos@gmail.com (K.A.); stamarios@yahoo.gr (M.S.); zkratiras@gmail.com (Z.K.); 2 Department of Microbiology, University Hospital of Ioannina, 45500 Ioannina, Greece; eleftheria.markou4@gmail.com; 3 Laboratory of Spermatology, Department of Urology, Faculty of Medicine, School of Health Sciences, University of Ioannina, 45110 Ioannina, Greece; azachariou@uoi.gr (A.Z.); nsofikit@uoi.gr (N.S.); 4 Department of Urology, Faculty of Medicine, School of Health Sciences, Aristotle University of Thessaloniki, 54124 Thessaloniki, Greece; helabio@yahoo.gr

**Keywords:** erectile dysfunction, gut microbiota, dysbiosis, inflammation, endothelial dysfunction, probiotics, treatment strategies

## Abstract

Erectile dysfunction (ED) is a prevalent male sexual disorder characterized by the persistent inability to achieve or maintain an erection sufficient for satisfactory sexual performance. While its etiology is multifactorial, encompassing vascular, neurological, hormonal, and psychological components, emerging evidence suggests a significant role for gut microbiota dysbiosis in its development. The gut microbiota influences various metabolic, inflammatory, and neuropsychological processes critical to erectile function. Dysbiosis can lead to systemic inflammation, endothelial dysfunction, hormonal imbalances, and altered neurotransmitter production, all of which are key factors in ED pathogenesis. This narrative review synthesizes current research on the association between gut microbiota alterations and ED, highlighting specific bacterial taxa implicated in ED through mechanisms involving inflammation, metabolic disturbances, and hormonal regulation. This review explores potential mechanisms linking gut microbiota and ED, including pro-inflammatory cytokines, gut barrier integrity disruption, metabolic disorders, psychological factors via the gut–brain axis, and hormonal regulation. Furthermore, the gut microbiota offers promising avenues for developing non-invasive biomarkers and therapeutic interventions such as probiotics, prebiotics, dietary modifications, and fecal microbiota transplantation. Future research should focus on longitudinal studies, mechanistic explorations, and clinical trials to validate these findings and translate them into clinical practice. Understanding the interplay between the gut microbiota and erectile function could unveil novel diagnostic biomarkers and pave the way for innovative treatments targeting the microbiota, ultimately improving men’s sexual and overall health.

## 1. Introduction

Erectile dysfunction (ED) is a prevalent male sexual disorder characterized by the persistent inability to achieve or maintain an erection sufficient for satisfactory sexual performance. It affects approximately 35–62% of men worldwide, with prevalence increasing significantly with age and the presence of comorbid conditions [1]. Notably, men over 60 years old exhibit a higher prevalence of ED, establishing aging as a significant predictor of the condition [2]. Comorbidities such as diabetes mellitus, hypertension, dyslipidemia, and obesity are well-documented contributors to ED, often leading to endothelial dysfunction and impaired vascular health, which are critical factors in its development [3].

Lifestyle factors also play a crucial role in ED risk. Smoking, sedentary behavior, and poor dietary habits are associated with an increased risk of ED, whereas regular physical activity and maintaining a healthy body weight offer protective benefits [4]. Additionally, psychological factors such as stress and depression can exacerbate or trigger ED, underscoring the multifactorial etiology of the condition [5].

The pathophysiology of ED is complex, involving endothelial dysfunction, impaired nitric oxide (NO) signaling, hormonal imbalances, and psychological factors [6]. Vascular health is paramount, as penile erection is fundamentally a vascular event driven by increased blood flow and the relaxation of smooth muscle within the corpora cavernosa [7]. Endothelial dysfunction reduces NO availability, impairing vasodilation and contributing to ED.

Although ED is multifactorial in etiology—with vascular, hormonal, and psychological factors traditionally taking center stage—the role of the gut microbiota has only recently garnered attention [8]. Emerging data suggest that gut dysbiosis can exacerbate systemic inflammation, metabolic disturbances, and even psychological stress—factors known to contribute to ED pathophysiology. As such, studying the gut microbiota in ED offers an underexplored avenue that may uncover novel diagnostic markers and therapeutic targets, potentially addressing root causes rather than merely symptomatic management [9].

Emerging evidence suggests that the gut microbiota may play a significant role in the development of ED. The gut microbiota, a complex ecosystem of trillions of microorganisms residing in the human gastrointestinal tract, including bacteria, archaea, viruses, and fungi, plays a pivotal role in maintaining human health by regulating metabolism, immune function, and neurobehavioral processes [10]. It aids in the digestion and fermentation of otherwise indigestible dietary fibers, producing short-chain fatty acids (SCFAs) like butyrate and acetate, which contribute to gut and systemic health by modulating inflammation and energy metabolism [11].

Dysbiosis, characterized by imbalances in the gut microbial community, has been associated with numerous chronic conditions such as metabolic syndrome, diabetes, inflammatory bowel disease (IBD), neurodegenerative disorders like Alzheimer’s disease, and male infertility [12,13,14]. These associations are mediated through the gut microbiota’s interactions with the host via mechanisms including SCFA production, the modulation of bile acid metabolism, and the influence on systemic immune responses [15]. Furthermore, the gut–brain axis, a bidirectional communication network between the gut and the central nervous system, highlights the gut microbiota’s role in influencing mood, cognition, and behavior through neuro-immunoendocrine pathways [16].

In the context of ED, dysbiosis may lead to systemic inflammation, endothelial dysfunction, and hormonal imbalances, all key factors in ED pathogenesis. For example, decreased production of SCFAs like butyrate can impair endothelial function by promoting inflammation and reducing NO availability. SCFAs are crucial in regulating inflammation, maintaining gut barrier integrity, and supporting the differentiation of colonic regulatory T cells, essential for immune tolerance [17].

Additionally, the modulation of bile acid metabolism by gut microbiota influences energy balance and immune homeostasis through interactions with bile acid receptors such as FXR and TGR5, which regulate inflammatory signaling pathways [18]. Tryptophan-derived microbial metabolites impact the aryl hydrocarbon receptor (AhR), enhancing epithelial barrier function and reducing gut inflammation [17].

The bidirectional relationship between the gut–brain axis and hormonal pathways further supports the potential role of gut microbiota in ED. Dysbiosis can lead to alterations in neurotransmitter production, immune activation, and hypothalamic–pituitary–adrenal (HPA) axis regulation, potentially affecting mood and stress levels, which are known to influence erectile function [19]. These processes are critical in maintaining vascular health and regulating sex hormones, both essential for erectile function [20].

Understanding the relationship between the gut microbiota and ED could provide insights into novel diagnostic biomarkers and therapeutic targets. This review aims to synthesize current research on the association between gut microbiota alterations and ED, explore the potential mechanisms linking them, and discuss future directions for research and therapeutic interventions. By elucidating the interplay between the gut microbiota and erectile function, we may unveil novel strategies for managing ED and improving men’s health.

## 2. Potential Mechanisms Linking Gut Microbiota and ED

### 2.1. Inflammation and Endothelial Dysfunction

Gut microbiota dysbiosis is strongly implicated in the activation of systemic inflammation, a key driver of endothelial dysfunction in ED. Dysbiosis compromises intestinal permeability, allowing bacterial endotoxins like lipopolysaccharide (LPS) to enter systemic circulation. LPS binds to Toll-like receptors (TLRs), particularly TLR4, on immune cells such as macrophages, triggering the release of pro-inflammatory cytokines including TNF-α, IL-6, and IL-1β [21]. These elevated cytokines increase oxidative stress and disrupt endothelial NO production, a critical factor for vasodilation and erectile function. Higher TNF-α levels correlate with worse erectile function scores, underscoring its role in ED pathophysiology [22].

A healthy gut barrier, maintained by tight junction proteins, is essential for preventing systemic exposure to harmful microbial products. Dysbiosis disrupts these tight junctions, resulting in “leaky gut” syndrome where LPS and microbial metabolites leak into the bloodstream, amplifying systemic inflammation [23]. This heightened inflammation is linked to endothelial dysfunction, atherosclerosis, and vascular complications commonly observed in ED patients. Chronic systemic inflammation exacerbates oxidative stress, damaging endothelial cells and reducing NO bioavailability, thereby impairing erectile capacity [24].

Additionally, inflammasomes, which are activated by bacterial metabolites such as LPS, play a role in ED pathogenesis. The activation of inflammasomes triggers caspase-1, leading to the maturation of IL-1β and IL-18. These cytokines intensify vascular inflammation and induce endothelial cell apoptosis, further aggravating ED symptoms [22].

### 2.2. Metabolic Disorders and ED

Metabolic disorders, including obesity, type 2 diabetes mellitus (T2DM), and hyperlipidemia, are strongly associated with ED due to their shared underlying mechanisms, such as insulin resistance, systemic inflammation, and endothelial dysfunction [25]. Recent findings have elucidated the role of gut microbiota in the pathogenesis of these metabolic conditions and their downstream effects on ED [10].

Dysbiosis significantly impacts host metabolism by altering energy harvest, fat storage, and glucose metabolism. A high *Firmicutes*-to-*Bacteroidetes* ratio has been consistently observed in obese individuals, contributing to increased caloric extraction from the diet and promoting weight gain [26]. Similarly, *Lachnospiraceae* and *Tyzzerella*, linked to high-fat diets, exacerbate metabolic dysfunction by promoting systemic inflammation and oxidative stress [27].

The gut microbiota influences key metabolic pathways by producing SCFAs like butyrate, propionate, and acetate through the fermentation of dietary fibers [28]. These SCFAs enhance insulin sensitivity, regulate lipid metabolism, and exert anti-inflammatory effects, offering protective benefits against metabolic diseases and their vascular complications [29]. However, in dysbiosis, SCFA production decreases, leading to compromised metabolic homeostasis and increased risk for ED.

Additionally, altered bile acid metabolism serves as a critical link between gut microbiota and metabolic health. Gut bacteria modify bile acids, influencing lipid absorption and cholesterol homeostasis. Dysbiosis-related changes in bile acid profiles can disrupt glucose regulation and exacerbate metabolic disorders [30]. Moreover, gut microbiota modulates incretins like glucagon-like peptide-1 (GLP-1), essential for glucose homeostasis [31]. Dysbiosis can impair incretin production, worsening insulin resistance and contributing to the pathophysiology of ED [32].

These findings highlight the intertwined roles of metabolic disorders and gut microbiota in ED. Further research is needed to identify specific microbial profiles and interventions that optimize gut microbiota for improved metabolic and sexual health outcomes.

### 2.3. Psychological Factors

Psychological factors play a critical role in ED, with the gut–brain axis emerging as a key mediator in the interplay between gut health and mental health. The gut–brain axis is a complex bidirectional communication network involving neural pathways (such as the vagus nerve), endocrine signaling, immune modulation, and metabolic interactions. Dysbiosis disrupts this axis, leading to alterations in neurotransmitter production, immune activation, and HPA axis regulation [33].

Depression and anxiety, common comorbidities in ED, are influenced by gut microbiota through several mechanisms. Gut bacteria produce neurotransmitters such as serotonin and gamma-aminobutyric acid (GABA), which modulate mood and cognitive functions. Dysbiosis reduces the production of these critical molecules, contributing to mood disorders [34]. Moreover, increased intestinal permeability, a hallmark of dysbiosis, allows LPS and other microbial products to enter systemic circulation, activating immune responses and releasing pro-inflammatory cytokines like IL-6 and TNF-α. These inflammatory mediators are linked to neuroinflammation and depression [35].

The HPA axis, a critical stress response system, is also modulated by the gut microbiota. Dysbiosis can hyperactivate the HPA axis, leading to elevated cortisol levels, which exacerbate stress and impair sexual function [36]. Studies have shown that specific microbial taxa, including *Lactobacillus* and *Bifidobacterium*, are associated with reduced HPA axis activation and improved psychological resilience. Conversely, increased levels of pathogenic bacteria such as *Bacteroides* and *Escherichia-Shigella* have been correlated with higher depression scores and anxiety, further linking dysbiosis to ED [37]. Modulating the gut microbiota may improve psychological well-being and, consequently, erectile function.

### 2.4. Hormonal Regulation

The gut microbiota plays a pivotal role in hormonal regulation, particularly in modulating sex hormone levels such as testosterone and estrogen. This relationship is mediated through the production of enzymes like β-glucuronidase, which deconjugate hormones in the gut, influencing their reabsorption and systemic levels. Dysbiosis can disrupt this intricate regulatory process, contributing to hormonal imbalances that impact ED [20].

Emerging evidence demonstrates the bidirectional interaction between gut microbiota and sex hormones. For instance, studies show that changes in gut microbial composition can directly affect testosterone levels. In animal models, gut microbiota transplantation from male to female hosts has been shown to increase testosterone levels, illustrating the gut’s capacity to influence endocrine function [38]. In humans, higher testosterone levels have been correlated with specific bacterial genera such as *Ruminococcus* and *Acinetobacter*, while reduced diversity in gut microbiota has been linked to hypogonadism, a condition commonly associated with ED [39].

The gut microbiota also interacts with the endocrine system to modulate metabolic pathways that influence sex hormone synthesis and activity. For example, gut microbes produce SCFAs and bile acid derivatives, which act as signaling molecules to regulate hormone receptor activity and inflammation. These metabolites are critical for maintaining the balance of androgens and estrogens, and their disruption can exacerbate conditions such as hypogonadism and vascular dysfunction linked to ED [40].

Dysbiosis also influences the HPA axis, a central regulator of reproductive hormones. This disruption can lead to altered feedback mechanisms, reduced testosterone production, and impaired libido and erectile function. Furthermore, systemic inflammation driven by gut-derived LPS has been shown to impair hormone signaling pathways, compounding the effects of ED [41].

These findings suggest that modulating the gut microbiota may offer therapeutic potential for restoring hormonal balance and improving ED outcomes. Future research is needed to explore the precise microbial species and pathways involved in this gut–hormone interaction to develop targeted interventions.

**Figure 1:**
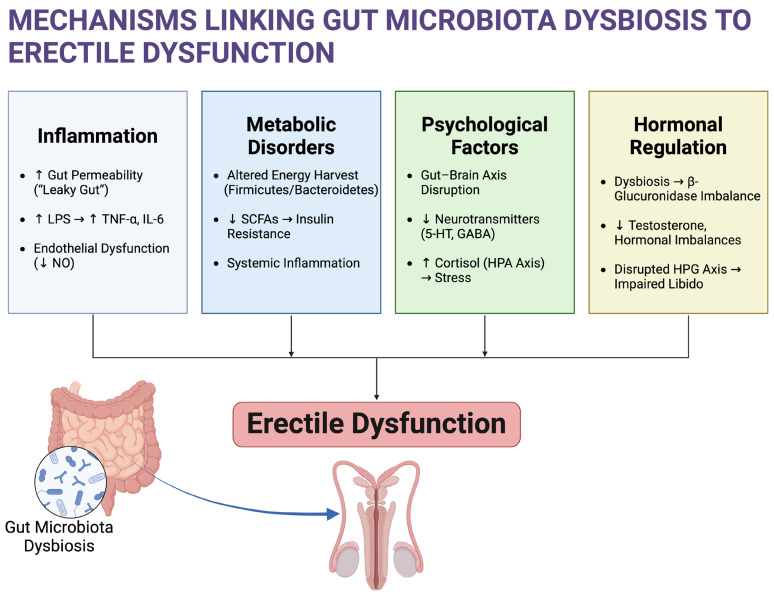
Mechanisms linking gut microbiota dysbiosis to erectile dysfunction. Created in BioRender. Kaltsas, A. (2025) https://BioRender.com/n85f897 (Effective Date: 17 January 2025). LPS: lipopolysaccharide; TNF-α: tumor necrosis factor alpha; IL-6: interleukin-6; NO: nitric oxide; SCFAs: short-chain fatty acids; 5-HT: serotonin (5-Hydroxytryptamine); GABA: gamma-aminobutyric acid; HPA axis: hypothalamic–pituitary–adrenal axis; HPG axis: hypothalamic–pituitary–gonadal axis.

To summarize the mechanisms discussed in this section, Figure 1 visually illustrates how gut microbiota dysbiosis contributes to ED. It integrates key pathways, including systemic inflammation, endothelial dysfunction, hormonal imbalances, and metabolic disturbances, highlighting their interconnected roles in ED pathogenesis.

## 3. Alterations in Gut Microbiota Composition in ED Patients

Recent studies have identified significant differences in the gut microbiota composition of men with ED compared to healthy controls, offering insights into the influence of gut dysbiosis in ED pathophysiology. These differences encompass microbial diversity, abundance, and specific taxa, underscoring the intricate relationship between the gut microbiome and systemic health [42,43,44]. The gut microbiota plays a critical role in modulating various physiological processes, including inflammation, metabolism, and vascular function, all of which are pertinent to the development and progression of ED [45,46].

### 3.1. Harmful Bacteria

The presence of certain harmful bacteria in the gut microbiota has been implicated in the pathophysiology of ED. This section will explore various genera of bacteria that have been associated with increased risk and severity of ED, focusing on their mechanisms of action and their contributions to systemic inflammation and endothelial dysfunction.

#### 3.1.1. *Actinomyces*

*Actinomyces*, a genus of Gram-positive, facultatively anaerobic bacteria, is commonly found in the oral cavity and gastrointestinal tract [47]. While often considered a commensal organism, *Actinomyces* can act as an opportunistic pathogen, causing diseases such as actinomycosis when mucosal barriers are breached [48]. Recent studies have identified its potential role in systemic conditions, including ED. A pilot study found that *Actinomyces* is significantly enriched in the gut microbiota of ED patients compared to healthy controls. The abundance of *Actinomyces* negatively correlates with erectile function metrics, such as nocturnal penile tumescence and rigidity (NPTR), indicating that higher levels are associated with worse erectile function [24].

*Actinomyces* may contribute to ED via its pro-inflammatory effects. Dysbiosis involving *Actinomyces* has been associated with increased levels of pro-inflammatory cytokines such as TNF-α and IL-6, which contribute to endothelial dysfunction [21]. Chronic inflammation impairs NO signaling, reducing the vasodilation necessary for erectile function. Beyond ED, elevated levels of *Actinomyces* have been linked to inflammatory diseases such as IBD, rheumatoid arthritis, and multiple sclerosis [49]. Elevated levels of inflammatory cytokines and prothrombotic compounds associated with *Actinomyces* may exacerbate vascular endothelial damage [50]. The pathogenic potential of *Actinomyces* in ED may involve its ability to translocate across compromised gut barriers, triggering systemic immune activation and endothelial injury. This pathway aligns with findings of reduced gut integrity and increased bacterial translocation in ED patients, further implicating microbial dysbiosis in the condition [51].

#### 3.1.2. *Bacteroides*

*Bacteroides* are opportunistic pathogens that have been found to be enriched in ED patients and are associated with increased systemic inflammation. These genera are known to translocate across compromised gut barriers, triggering inflammatory responses that impair vascular function and contribute to ED. Their abundance is often linked to diets high in saturated fats and low in fiber, underscoring the role of diet in modulating gut microbiota and ED risk [21]. The presence of *Bacteroides* in the gut microbiome has been correlated with elevated levels of inflammatory markers, which can lead to endothelial dysfunction [52].

#### 3.1.3. *Clostridium XVIII*

*Clostridium XVIII*, a genus within the Clostridia class of the Firmicutes phylum, plays a role in immune modulation and gut health. Studies suggest its relevance to ED through interactions with inflammatory pathways and gut microbiota dysbiosis [53]. *Clostridium XVIII* abundance is significantly higher in men with lower erectile function, as measured by the International Index of Erectile Function-5 (IIEF-5). In a community-based study, its relative abundance was an independent factor associated with lower IIEF-5 scores, suggesting a potential link to ED pathogenesis [21].

*Clostridium XVIII* influences immune responses by promoting regulatory T cells (Tregs) through the production of SCFAs, including butyrate. This modulation of inflammatory pathways and maintenance of immune homeostasis are crucial in controlling systemic inflammation, a key contributor to endothelial dysfunction and vascular impairment central to ED [54]. Additionally, *Clostridium XVIII* is associated with bowel movement disorders such as constipation and diarrhea, which are often seen in metabolic diseases. Dysbiosis involving *Clostridium XVIII* has been linked to elevated pro-inflammatory cytokine levels, including TNF-α and IL-6, contributing to endothelial damage and reduced NO availability [55]. However, the precise role of *Clostridium XVIII* in ED remains unclear, and further research is needed to elucidate its impact on vascular and immune functions.

#### 3.1.4. *Escherichia*/*Shigella*

*Escherichia/Shigella*, a genus within the Proteobacteria phylum, has garnered significant attention for its role in the pathophysiology of ED through its association with systemic inflammation and endothelial dysfunction. Elevated levels of *Escherichia/Shigella* have been observed in conditions of gut dysbiosis, where the genus contributes to the disruption of gut homeostasis and promotes pro-inflammatory cytokine production, including TNF-α and IL-6. These cytokines interfere with endothelial integrity, impairing NO bioavailability, which is essential for vascular relaxation and erectile function [56]. Moreover, *Escherichia/Shigella* produces LPSs, potent inflammatory agents that exacerbate vascular oxidative stress, a critical pathway in the pathogenesis of ED [57]. These findings underscore the genus’s ability to mediate a cascade of immune and vascular disturbances, further linking gut microbiota imbalance to the systemic vascular complications characteristic of ED [58].

Beyond its inflammatory effects, *Escherichia/Shigella* also influences metabolic and hormonal pathways that are critical to erectile function. The genus has been implicated in the disruption of metabolic homeostasis, including insulin resistance and glucose dysregulation, both of which are established risk factors for ED [59]. Additionally, *Escherichia/Shigella* may impair testosterone synthesis and endocrine balance, further compounding its impact on sexual health. LPS from this genus is known to activate inducible nitric oxide synthase (iNOS), increasing oxidative stress and further amplifying inflammatory and metabolic pathways involved in ED [60]. These multifaceted mechanisms suggest that targeted interventions addressing gut dysbiosis and *Escherichia/Shigella* overgrowth may hold promise in mitigating the adverse effects of this bacterial genus on vascular and metabolic health.

#### 3.1.5. *Fusobacterium*

*Fusobacterium* species have been increasingly recognized as significant contributors to systemic inflammation and various health complications, particularly in patients with ED. The enrichment of *Fusobacterium* in the gut microbiota is associated with compromised gut barrier integrity, leading to bacterial translocation into the bloodstream. This translocation triggers inflammatory responses that can impair vascular function, a critical factor in the pathophysiology of ED. Studies have shown that *Fusobacterium* can induce a proinflammatory microenvironment, which may exacerbate vascular dysfunction through mechanisms involving immune cell death and alterations in endothelial integrity [61,62]. Furthermore, the presence of *Fusobacterium* has been linked to increased levels of inflammatory markers, such as LPS, which correlate with systemic inflammation and may contribute to the development of metabolic and cardiovascular comorbidities often observed in ED patients [63,64].

#### 3.1.6. *Oscillibacter*

The genus *Oscillibacter* has been positively associated with the risk of ED. This genus is linked to pro-inflammatory states and metabolic disorders, including insulin resistance and obesity, which are established risk factors for ED. Additionally, *Oscillibacter* is connected to psychological conditions such as depression, often co-occurring with ED, thereby compounding its effects on erectile function [65].

#### 3.1.7. *Streptococcus*

The genus *Streptococcus* is enriched in ED patients and is strongly associated with pro-inflammatory states. *Streptococcus* species are known to increase levels of pro-inflammatory cytokines such as TNF-α and IL-6, which disrupt endothelial function by impairing NO signaling and promoting vascular damage [66,67].

Additionally, *Streptococcus* has been linked to systemic diseases that involve inflammatory pathways, such as cardiovascular disorders. Its elevated abundance in ED patients suggests a role in driving endothelial dysfunction and exacerbating vascular inflammation critical in the pathogenesis of ED [68].

#### 3.1.8. *Tyzzerella*

*Tyzzerella* has been identified as another genus with increased abundance in ED patients. Its presence is associated with a heightened risk of cardiovascular disease (CVD) and metabolic syndrome. Elevated levels of *Tyzzerella* may exacerbate systemic inflammation and oxidative stress, further impairing vascular health [51].

### 3.2. Beneficial Bacteria

#### 3.2.1. *Alistipes*

*Alistipes*, a genus of anaerobic, Gram-negative bacteria within the Bacteroidetes phylum, has emerged as a significant player in inflammation modulation and gut health. Recent studies have observed a significant reduction in *Alistipes* abundance in individuals with ED, linking this bacterial genus to critical vascular and inflammatory pathways involved in erectile function [21].

This genus produces bioactive compounds, including sulfonolipids, which act as antagonists to the von Willebrand factor receptor. This antagonism suppresses the production of pro-inflammatory cytokines like TNF-α and IL-6, which are implicated in endothelial dysfunction and vascular impairment associated with ED [69]. Furthermore, *Alistipes* contributes to maintaining gut barrier integrity and exerts systemic anti-inflammatory effects, offering protection against ED-related vascular damage.

#### 3.2.2. *Coprococcus*

The genus *Coprococcus*, part of the *Lachnospiraceae* family, is significantly reduced in ED patients. This decreased abundance correlates with a higher prevalence of metabolic diseases such as T2DM, hyperlipidemia, and obesity [24]. *Coprococcus* contributes to gut health by producing butyrate, an SCFA that exerts anti-inflammatory effects and supports endothelial function, thereby protecting against systemic inflammation and endothelial dysfunction central to ED development.

#### 3.2.3. *Lactococcus*

*Lactococcus*, recognized for its probiotic potential, is depleted in ED patients. This genus is vital for maintaining gut barrier integrity and modulating the immune response. A reduction in *Lactococcus* compromises these protective mechanisms, resulting in increased gut permeability, systemic inflammation, and vascular impairments associated with ED [51].

#### 3.2.4. *Lachnospiraceae*

The *Lachnospiraceae* family, comprising anaerobic, Gram-positive bacteria within the Firmicutes phylum, plays a pivotal role in gut homeostasis by fermenting complex carbohydrates to produce SCFAs, particularly butyrate. Butyrate has well-documented anti-inflammatory properties and contributes to gut barrier integrity and systemic metabolic regulation [70].

Studies on *Lachnospiraceae* in ED have yielded mixed findings. A Mendelian randomization analysis by Su et al. (2023) reported that an increased abundance of *Lachnospiraceae* is associated with higher odds of ED (OR: 1.27), suggesting that certain taxa within this family may influence blood lipid levels and neurotransmission pathways, thereby contributing to ED pathophysiology. Conversely, specific taxa within Lachnospiraceae that are butyrate producers, such as Roseburia, exhibit protective metabolic effects [70].

*Roseburia* is a prominent butyrate-producing genus within this family. Its abundance is significantly reduced in ED patients, contributing to elevated systemic inflammation, impaired gut barrier integrity, and endothelial dysfunction. Butyrate produced by *Roseburia* mitigates oxidative stress and supports NO bioavailability, a crucial mediator of penile vasodilation and vascular health [68].

The heterogeneity within *Lachnospiraceae* likely accounts for the conflicting observations regarding its role in ED. Certain genera, such as *Lachnospiraceae NC2004*, are associated with pro-inflammatory states and metabolic dysfunctions, increasing the risk of ED [71]. In contrast, butyrate-producing genera like Roseburia contribute to reduced systemic inflammation, improved glucose metabolism, and enhanced gut barrier function, offering protective effects against ED [72].

#### 3.2.5. *Ruminococcaceae*

The *Ruminococcaceae* family, within the Firmicutes phylum, is integral to gut health, primarily through fermenting dietary fibers into SCFAs like butyrate. Butyrate is renowned for its anti-inflammatory properties, maintenance of gut barrier integrity, and promotion of metabolic health. Notably, *Ruminococcaceae UCG-013* has been identified as potentially protective against ED [65].

Increased abundance of *Ruminococcaceae UCG-013* correlates with a decreased risk of ED, likely due to enhanced butyrate production. This SCFA modulates systemic inflammation and oxidative stress, which are key factors in ED pathogenesis [73]. By reducing inflammation and oxidative damage, butyrate-producing bacteria such as *Ruminococcaceae UCG-013* improve endothelial function, thereby enhancing vascular health and erectile capacity [71].

It remains challenging to distinguish whether observed microbial signatures in ED patients are causative or associative. Most current studies rely on cross-sectional data, which limit causal inference. Factors such as dietary habits, medication use, and comorbid conditions can independently alter the gut microbiota and confound results. Future longitudinal studies and mechanistic investigations, including germ-free animal models and Mendelian randomization analyses, will be essential for clarifying whether specific microbial taxa drive ED pathophysiology or simply reflect concurrent metabolic and inflammatory states [74].

**Figure 2:**
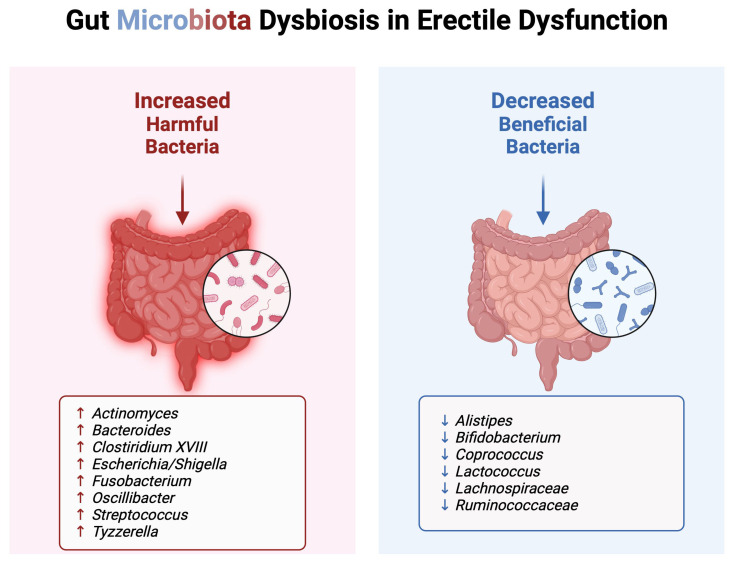
Gut microbiota alterations in erectile dysfunction. Created in BioRender. Kaltsas, A. (2025) https://BioRender.com/g09f030 (Effective Date: 15 January 2025).

Figure 2 provides a visual representation of the alterations in gut microbiota composition in ED patients. It highlights the increased abundance of harmful bacteria and the decreased presence of beneficial bacteria associated with unhealthy dietary patterns.

### 3.3. Consideration of Covariables and Summary of Key Taxa

Although multiple studies have reported associations between specific gut microbial taxa and ED, most existing research is cross-sectional or relies on small pilot samples [21,24,75]. These designs, while valuable for preliminary insights, often do not uniformly control for potential confounders such as nutritional status, medication use (including antibiotics or proton pump inhibitors), and comorbid conditions (e.g., obesity, diabetes, hypertension), all of which can profoundly affect the gut microbiota [23,71].

For instance, Okamoto et al. conducted a community-based cross-sectional analysis of Japanese men to link gut dysbiosis with ED severity but did not provide detailed data on participants’ dietary intake or body mass index (BMI) [21]. Similarly, Kang et al. identified correlations between specific bacterial genera (e.g., *Actinomyces* and *Ruminococcaceae*) and erectile function in a Chinese cohort, yet only basic demographic and inflammatory markers were accounted for [24]. Some other investigations reported microbial shifts in ED patients without rigorously adjusting for comorbid metabolic disorders [42,65].

Failing to consider these covariables may overestimate or underestimate the true relationship between gut microbial alterations and ED risk. Future research should adopt multivariable approaches—for example, controlling for BMI, dietary composition, medication history, and metabolic status—to better isolate the microbiome’s specific role in ED pathogenesis. Additionally, longitudinal and interventional designs (e.g., probiotic supplementation trials, fecal microbiota transplantation studies) are essential to distinguish causation from correlation and elucidate mechanistic pathways.

To offer a consolidated overview of the most commonly implicated bacterial genera and their potential impact on erectile function, Table 1 outlines the primary taxa reported across different studies, along with their associated metabolites, mechanistic pathways, and supporting references.

## 4. Potential Biomarkers and Diagnostic Tools

The gut microbiota offers a promising avenue for the development of non-invasive biomarkers for ED. Several studies have consistently identified specific bacterial taxa that are differentially abundant in ED patients compared to healthy controls. For instance, the enrichment of *Actinomyces* and the depletion of beneficial taxa like *Ruminococcaceae UCG-013* are strongly correlated with reduced erectile function, making these taxa potential biomarkers for ED diagnosis [24]. Similarly, taxa such as *Alistipes* and *Clostridium XVIII* have shown significant associations with ED and may serve as indicators of disease progression [21].

Machine learning algorithms are emerging as powerful tools in leveraging microbiota data for diagnostic purposes. Predictive models incorporating microbiota profiles have demonstrated high diagnostic accuracy. For example, a random forest model based on gut microbial abundance achieved an area under the curve (AUC) of 0.72 in identifying ED, suggesting that gut microbiota can be integrated into predictive frameworks for early and precise diagnosis [24]. Another study utilized Mendelian randomization to confirm causal relationships between certain taxa, such as *Lachnospiraceae* and *Oscillibacter*, and ED, further supporting their potential utility as biomarkers [65].

Despite the promise of these biomarkers, several challenges remain. Inter-individual variability in gut microbiota composition due to genetic, dietary, and environmental factors can complicate biomarker standardization. Additionally, differences in sampling, sequencing methodologies, and bioinformatics pipelines across studies can influence results, necessitating standardized protocols [71]. Methodological heterogeneity in microbiome research poses a significant challenge. Variability in sample collection (e.g., stool vs. mucosal swabs), storage conditions, DNA extraction kits, and sequencing platforms (16S rRNA vs. shotgun metagenomics) can lead to inconsistent findings across studies. Furthermore, inter-individual differences in diet, genetics, and medication use (particularly antibiotics and proton pump inhibitors) can mask disease-specific microbial signatures. Establishing standardized protocols for sample collection, sequencing pipelines, and data analysis will be critical in producing reproducible and comparable data that can reliably inform ED-related microbiome research [76,77].

The longitudinal stability of microbiota-based biomarkers also poses a challenge, as the gut microbiome can be dynamic over time and affected by factors like medication and diet [75]. Future directions include the validation of gut microbiota-based biomarkers in large, multicenter cohorts and the integration of microbiota data with other clinical variables to enhance diagnostic precision. Advances in machine learning and omics technologies hold the potential to refine these tools further, enabling personalized interventions based on microbiota profiles. By addressing the current limitations and advancing research, gut microbiota could revolutionize the early diagnosis and management of ED.

## 5. Therapeutic Implications

The modulation of gut microbiota has emerged as a promising therapeutic avenue for managing ED, leveraging the interconnectedness of the microbiome with systemic inflammation, metabolism, and vascular health. Recent advances in probiotics, prebiotics, dietary interventions, and fecal microbiota transplantation (FMT) underscore their potential to restore microbial balance and improve erectile function.

Probiotic supplementation, particularly with strains like *Lactobacillus* and *Bifidobacterium*, has shown benefits in reducing systemic inflammation and enhancing gut barrier integrity, key factors in mitigating ED. These bacteria promote the production of SCFAs, such as butyrate, which are known to exert anti-inflammatory effects, regulate lipid metabolism, and support endothelial health. In animal models, probiotics have been associated with increased testosterone levels and improved vascular function, suggesting a direct link to sexual health [78]. While the field is still evolving, several probiotic strains—particularly those within *Lactobacillus* (e.g., *L. reuteri*, *L. rhamnosus*) and *Bifidobacterium* (e.g., *B. animalis*)—show promise in reducing systemic inflammation and enhancing endothelial function in animal models [79,80]. Preliminary human trials in metabolic syndrome populations have also hinted at improvements in biomarkers related to erectile function, though specific ED-focused studies are needed [81]. Prioritizing bacterial taxa for targeted intervention may be guided by their known roles in SCFA production (e.g., *Ruminococcaceae*, *Lachnospiraceae*) and immune modulation (e.g., *Bifidobacterium*, *Lactobacillus*) [82]. Similarly, prebiotics, which are non-digestible fibers that promote the growth of beneficial microbes, enhance SCFA production and gut microbial diversity, potentially improving metabolic and vascular outcomes relevant to ED [83].

To date, only a small number of observational and pilot studies have explored gut microbiota-targeted strategies in ED or closely related conditions. Table 2 summarizes the key human studies, highlighting their designs, interventions, and outcome measures.

Dietary modifications, including increased intake of fiber-rich foods like fruits, vegetables, and whole grains, have been shown to favorably alter gut microbiota composition, reducing the abundance of pro-inflammatory taxa and enhancing the growth of beneficial microbes. Diets low in saturated fats and processed foods have also been associated with improved gut barrier integrity and reduced systemic inflammation, both of which are critical for vascular health and erectile capacity [84].

FMT is an emerging approach that involves the transfer of a healthy donor’s gut microbiota to restore microbial balance in the recipient. While FMT is primarily used to treat gastrointestinal disorders like *Clostridium difficile* infection, its potential to improve systemic inflammation and metabolic parameters has garnered attention in other conditions, including ED. FMT has been shown to positively influence microbial diversity and reduce inflammation, offering a potential avenue for treating ED, particularly in patients with comorbid metabolic syndrome or obesity [85].

Future strategies may include engineered probiotics or bacteriophage-based therapies for the targeted manipulation of specific microbial populations, providing precision therapy that addresses the unique dysbiosis linked to ED. However, further research is necessary to understand the long-term safety and efficacy of these approaches. In this context, personalized interventions founded on microbiome profiles could pave the way for truly individualized treatments. Future clinical trials should therefore adopt a precision-medicine approach, tailoring probiotic and prebiotic formulations based on the patient’s baseline microbiota profile, comorbidities, and dietary patterns, ultimately enhancing the management of ED and associated conditions.

Overall, therapeutic modulation of the gut microbiota represents a novel and integrative approach to improving erectile function, with significant implications for systemic health. Continued investigation and larger clinical trials are essential to validate these strategies and optimize their application in clinical practice.

## 6. Future Directions in Treatment Strategies

Future treatment strategies for ED through gut microbiota modulation represent an exciting and evolving area of research. Key priorities include identifying therapeutic targets such as specific bacterial strains or microbial metabolites that influence erectile function. Recent advances highlight the potential of taxa like *Ruminococcaceae* and *Lactobacillus*, as well as metabolites like butyrate, in reducing systemic inflammation and improving vascular health. However, further studies are needed to pinpoint and validate these targets in diverse populations [24].

Understanding the mechanisms by which the gut microbiota influences ED is essential. Pathways involving inflammation, endocrine disruption, and the gut–brain axis have been implicated, but more robust mechanistic studies are required to delineate these interactions. Animal models and advanced technologies like multi-omics and microbial genome editing could unravel the causal pathways, offering insights into novel therapeutic approaches [20].

Personalized medicine presents a promising direction in ED management, with the potential to tailor treatments based on an individual’s gut microbiota profile, metabolic status, and genetic predispositions. Integrating microbiome data with genomic, metabolomic, and clinical parameters could lead to precision therapies. For instance, patients with a specific microbial imbalance may benefit from targeted probiotic or prebiotic formulations, while others might require dietary adjustments or innovative treatments like bacteriophage therapy [71].

Clinical trials evaluating the efficacy and safety of microbiota-based interventions are urgently needed. Randomized controlled trials (RCTs) with adequate sample sizes and long follow-up periods will be instrumental in validating these therapies. Preliminary studies on probiotics and FMT have shown promise, but larger-scale trials are necessary to establish their role alongside conventional ED treatments like phosphodiesterase-5 inhibitors [65,86].

The limitations of current research must also be addressed to advance the field. Many studies suffer from small sample sizes and lack diversity, limiting the generalizability of findings. Ethnicity, diet, and environmental factors significantly influence gut microbiota, underscoring the need for multicenter studies with heterogeneous cohorts. Methodological inconsistencies, including differences in sequencing techniques, sample handling, and data analysis pipelines, pose another challenge. Standardized protocols are critical for generating reliable and comparable data [23].

Most existing studies are cross-sectional, which precludes the establishment of causality. Longitudinal studies tracking microbiota changes over time, combined with mechanistic research using animal models, are necessary to identify causal relationships and assess the dynamic effects of interventions. Confounding factors, such as age, comorbidities, medications, and lifestyle behaviors, must be rigorously controlled in future research to isolate the specific contributions of the gut microbiota to ED [75].

Finally, combining gut microbiota modulation with conventional ED treatments may offer synergistic benefits by addressing both the symptoms and underlying pathophysiological mechanisms. Collaborative efforts integrating microbiome science, endocrinology, urology, and bioinformatics will be essential to transform microbiota-based therapies from concept to clinical reality. Future studies should aim not only to improve ED outcomes but also to enhance overall health, recognizing the interconnected nature of the microbiome with systemic physiology.

## 7. Conclusions

Emerging evidence underscores a significant association between gut microbiota dysbiosis and ED, implicating specific bacterial taxa such as *Actinomyces*, *Lachnospiraceae*, *Ruminococcaceae UCG-013*, and *Alistipes* in its pathogenesis. These microorganisms influence erectile function through multiple mechanisms, including systemic inflammation, endothelial dysfunction, metabolic disturbances, hormonal regulation, and psychological factors mediated via the gut–brain axis. The complex interplay between gut microbiota and host physiology suggests that dysbiosis contributes to the multifactorial etiology of ED.

Despite these promising insights, current research remains in its early stages, necessitating more rigorous and longitudinal studies to validate findings and establish causality. Methodological inconsistencies, small sample sizes, and heterogeneity among study populations pose significant challenges to generalizing results. Modulating the gut microbiota presents a novel therapeutic avenue for ED, offering hope for innovative, non-invasive treatments. Future research should focus on mechanistic explorations, standardized methodologies, and well-designed clinical trials to harness the therapeutic potential of the gut microbiome. Integrating microbiome-based interventions with conventional therapies may enhance treatment efficacy, ultimately improving men’s sexual and overall health.

## Figures and Tables

**Table 1:** Summary of selected bacterial genera, their associated metabolites, and proposed effects on erectile dysfunction.

Bacterial Genus/Family	Key Metabolites	Proposed Role in ED	Mechanistic Pathway(s)	References
*Actinomyces*	Unknown or LPS-like components	- Enriched in ED patients; negatively correlates with erectile function scores - Elevates TNF-α, IL-6, contributing to endothelial dysfunction	Inflammation, endothelial injury, possible gut barrier disruption	[24,47,48,49,50,51]
*Bacteroides*	Lipopolysaccharide (LPS), bile acid metabolites	- Associated with increased systemic inflammation and vascular impairment - Often linked to diets high in saturated fats	Inflammation, gut barrier compromise	[21,52]
*Escherichia/Shigella*	LPS, pro-inflammatory metabolites	- Elevates TNF-α, IL-6, impairing NO availability - Contributes to oxidative stress, potentially disrupting hormone balance	Inflammation, oxidative stress, metabolic/hormonal dysregulation	[56,57,58,59,60]
*Fusobacterium*	LPS, other inflammatory mediators	- Linked to compromised gut barrier, increased bacterial translocation - Induces vascular dysfunction and immune cell death	Inflammation, gut barrier disruption	[61,62,63,64]
*Oscillibacter*	Short-chain fatty acids (SCFAs; e.g., butyrate)	- Positively associated with ED risk - Linked to pro-inflammatory states, metabolic issues (insulin resistance, obesity)	Inflammation, metabolic dysregulation, possible mood interactions	[65]
*Streptococcus*	Possibly LPS-like components, other virulence factors	- Raises TNF-α, IL-6, disrupting NO signaling - Linked to cardiovascular disorders and vascular inflammation	Inflammation, endothelial dysfunction	[66,67,68]
*Tyzzerella*	Pro-inflammatory metabolites (not fully characterized)	- Elevated in ED, associated with CVD and metabolic syndrome - May exacerbate oxidative stress, impairing vascular health	Inflammation, oxidative stress	[51]
*Alistipes*	Sulfonolipids, SCFAs (e.g., propionate, butyrate)	- Reduced in ED patients; exerts anti-inflammatory, gut-protective effects - May inhibit TNF-α, IL-6 via von Willebrand factor receptor antagonism	Anti-inflammation, gut barrier support	[21,69]
*Coprococcus*	Butyrate and other SCFAs	- Decreased in ED; correlates with reduced metabolic and vascular complications - Supports endothelial function by mitigating inflammation	Anti-inflammation, endothelial support	[24]
*Lactococcus*	SCFAs, bacteriocins, lactate	- Depleted in ED; helps maintain gut barrier and lower systemic inflammation - Reduced levels linked to increased gut permeability and vascular risk	Gut barrier integrity, immune modulation	[51]
*Lachnospiraceae*	SCFAs (butyrate, propionate), varies by genus	- Some taxa (e.g., *Roseburia*) produce butyrate (anti-inflammatory), others linked to pro-inflammatory states - Disruption can affect lipid metabolism, endocrine function, and NO bioavailability	Inflammation, lipid metabolism, NO signaling	[70,71,72]
*Ruminococcaceae (UCG-013)*	SCFAs (especially butyrate)	- Increased abundance inversely linked to ED risk - Enhances endothelial function by reducing inflammation and oxidative damage	Anti-inflammation, endothelial protection	[65,71,73]

ED, erectile dysfunction; NO, nitric oxide; LPS, lipopolysaccharide; SCFA, short-chain fatty acid; TNF-α, tumor necrosis factor-alpha; IL-6, interleukin-6; CVD, cardiovascular disease.

**Table 2:** Summary of key human studies investigating gut microbiota and erectile dysfunction.

Study (Year)	Study Design/Population	Intervention/Exposure	Outcome Measures	Key Findings	References
Okamoto et al. (2020)	Cross-sectional, community-based study in Japan (n = 68 men)	No intervention; fecal samples collected for microbiota profiling	Prevalence of ED, gut microbial composition	Enrichment of *Bacteroides* and *Escherichia/Shigella* associated with higher ED severity. Study suggests a possible link between dysbiosis and ED.	[21]
Su et al. (2023)	Mendelian randomization analysis (population-level genetic data)	Genetic proxies for gut microbiota traits	Causal relationship between certain taxa and ED	*Lachnospiraceae* and *Oscillibacter* variants showed potential causal links to ED risk, providing genetic evidence for gut microbiota’s role in ED pathogenesis.	[65]
Kang et al. (2024)	Pilot observational study in China (n = 36 ED patients vs. 36 controls)	No intervention; 16S rRNA gene sequencing of stool samples	IIEF-5 scores, gut microbiota composition, inflammatory markers	ED group showed increased abundance of *Actinomyces*, decreased *Ruminococcaceae* spp. Negative correlation between *Actinomyces* load and erectile function.	[24]
Osman et al. (2024)	Case–control pilot study in the USA (n = 19 ED patients vs. 19 controls)	No intervention; stool sample analysis of microbial composition	Gut microbiota composition, ED severity	Identified differences in gut microbial diversity; suggested that specific taxa might influence inflammatory pathways contributing to ED.	[75]
Zhu et al. (2024)	Narrative synthesis of gut microbiota and ED studies	No direct intervention; overview of observational findings	ED-associated changes in gut microbiome	Summarized cross-sectional data linking dysbiosis to ED, highlighting potential inflammatory and metabolic pathways. Called for larger RCTs to confirm efficacy.	[9]

ED, erectile dysfunction; IIEF-5, International Index of Erectile Function-5; RCT, randomized controlled trial.
